# Lattice Boltzmann Solver for Multiphase Flows: Application to High Weber and Reynolds Numbers

**DOI:** 10.3390/e23020166

**Published:** 2021-01-29

**Authors:** Seyed Ali Hosseini, Hesameddin Safari, Dominique Thevenin

**Affiliations:** 1 Laboratory of Fluid Dynamics and Technical Flows, University of Magdeburg “Otto von Guericke”, D-39106 Magdeburg, Germany; hesameddin.safari@ovgu.de (H.S.); thevenin@ovgu.de (D.T.); 2 Department of Mechanical and Process Engineering, ETH Zürich, 8092 Zürich, Switzerland

**Keywords:** lattice Boltzmann method, multiphase flows, conservative Allen–Cahn, phase field

## Abstract

The lattice Boltzmann method, now widely used for a variety of applications, has also been extended to model multiphase flows through different formulations. While already applied to many different configurations in low Weber and Reynolds number regimes, applications to higher Weber/Reynolds numbers or larger density/viscosity ratios are still the topic of active research. In this study, through a combination of a decoupled phase-field formulation—the conservative Allen–Cahn equation—and a cumulant-based collision operator for a low-Mach pressure-based flow solver, we present an algorithm that can be used for higher Reynolds/Weber numbers. The algorithm was validated through a variety of test cases, starting with the Rayleigh–Taylor instability in both 2D and 3D, followed by the impact of a droplet on a liquid sheet. In all simulations, the solver correctly captured the flow dynamics andmatched reference results very well. As the final test case, the solver was used to model droplet splashing on a thin liquid sheet in 3D with a density ratio of 1000 and kinematic viscosity ratio of 15, matching the water/air system at We = 8000 and Re = 1000. Results showed that the solver correctly captured the fingering instabilities at the crown rim and their subsequent breakup, in agreement with experimental and numerical observations reported in the literature.

## 1. Introduction

The lattice Boltzmann method (LBM) is a discrete solver for the so-called discrete velocity Boltzmann equation (DVBE), initially developed as an alternative to classical solvers for the incompressible hydrodynamic regime [1,2]. Due to the simplicity of the algorithm, low computational cost of discrete time-evolution equations, and locality of nonlinear terms and boundary conditions, it has rapidly grown over the past few decades [3]. While intended for the incompressible regime, the LBM formally solves the compressible isothermal Navier-Stokes (NS) equations at a reference temperature. While originally tied to the considered flow’s temperature, in the context of the lattice Boltzmann (LB) solver, the reference temperature is a numerical parameter allowing for control over convergence and consistency [1]. Weak compressibility in the formulation along with the parabolic nature of the partial differential equation (PDE) governing the evolution of pressure, as opposed to Chorin’s original artificial compressibility method (ACM), made the scheme efficient and applicable to unsteady flows [4]. Although originally used for single-phase flows, it has since been extended to multiphase, multispecies, and compressible flows.

While generally based on diffuse-interface formulations, LB solvers for multiphase flows can be categorized as pertaining to one of three major categories: (a) pseudopotential [5,6], (b) free energy [7,8], and (c) phase field. Other types of formulations can also be found in the literature, but they are not as widely spread and/or developed as these three.

In the context of the free-energy formulation, the expression for the nonlocal nonideal pressure tensor is found through the free-energy functional. The appropriate pressure tensor is then introduced into the LB solver via a moment-matching approach assigning coefficients to different terms in the equilibrium distribution function (EDF) [8]. This formulation is consistent and differentiated from the generic double-well potential-based Cahn–Hilliard formulation because, in the minimization process of free energy, the equation of state (EoS) is explicitly considered. As is the case for the pseudopotential formulation, the explicit intervention of the EoS within the free functional ties the thickness of the interface to physical parameters, e.g., surface tension, density ratio, and EoS. As a consequence, the choice of the EoS and/or tuning of the coefficients in the EoS is a method of choice to widen the area of accessible density ratios. This approach was later extended by introducing nonideal components of the pressure tensor via external body forces. Introducing these effects with a body force made the scheme more stable by reducing Galilean invariance issues tied to the third-order moments of the EDF [9].

The pseudopotential formulation follows more of a bottom–up approach in introducing nonideal dynamics into the solver. It follows the general philosophy of the Boltzmann–Vlasov equation, introducing a nonlocal potential to account for nonideal effects. While the original formulation relied on what was termed effective density, actual EoS were introduced into the pseudopotential in [10,11]. Apart from thermodynamic consistency, the possibility of using different EoS allowed for higher density ratios to be modelled. As the free-energy formulation, this model is limited to lower Weber number regimes because it naturally comes with large surface-tension values. While more advanced models allow for the independent tuning of surface tension [12], the spectrum of values covered by the model is rather limited and barely allows for variations of one order of magnitude [13].

The last category is based on the free-energy functional minimization approach, just like the free-energy approach. However, contrary to the latter, the surface and bulk energies used in the minimization process are those of a generic double-well potential [14], allowing for decoupling, among other parameters, the interface thickness from the fluid physical properties. Another consequence of this choice of functional is the partial loss of thermodynamic consistency, making the extension of the formulation to more complex physics such as thermal flows, compressible flows and acoustics less straightforward, although a number of attempts were documented in the literature [15,16,17]. Nevertheless, it was observed to be very effective and robust for multiphase flows in the incompressible regime, and readily able to deal with larger Weber numbers. For a more comprehensive overview of the developments of such models, interested readers are referred to [18]. Approaches relying on the explicit tracking of the interface with a consistent energy functional making use of the nonideal EoS were also proposed as ways to improve the stability of the original free-energy formulation [19,20].

Over the past few decades, much effort has been put into developing phase-field-based LB solvers for various applications [16,21,22]. Given that in such formulations local density is a dependent variable on the local value of the order parameter, they have to be coupled to a modified form of the LB solver for the flow usually referred to as incompressible formulation. The so-called low-Mach formulation is mostly based on the modified distribution function introduced in [19], where pressure is the zeroth-order moment of the distribution function. This flow solver was combined with different forms of interface-tracking formulations, e.g., Allen-Cahn (AC), conservative AC, or Cahn-Hilliard (CH) to model multiphase flows. The aim of the present study is to introduce a multiphase solver relying on the pressure-based formulation of [19] and a multiple relaxation time (MRT) realization for the flow solver coupled with a LB solver for the conservative AC. The use of the MRT collision operator in cumulant space with the decoupled interface tracking allows for simulations in high Reynolds and Weber regimes. After a brief introduction of the model, it is used to simulate a variety of test cases, proving its ability to reproduce correct physics and its robustness. All models were implemented in our in-house multiphysics solver, ALBORZ [23].

## 2. Theoretical Background

### 2.1. Target Macrosopic System

As briefly stated in the introduction, the aim of the present work is to solve multiphase flow equations within the context of the diffuse interface formulation in the limit of an incompressible regime, where interface dynamics are followed and accounted for via an additional indicator field, ϕ. As such, at the macroscopic level, low Mach NS equations are targeted:

(1) $$\partial_{t}\rho u_{i}+\partial_{j}\rho u_{i}u_{j}+\partial_{j}\sigma_{ij}+\mu_{\phi }\partial_{i}\phi +F_{b,i}=0,$$

where $u_{i}$ is fluid velocity, ρ the fluid density, and $F_{b,i}$ designates external body forces. The stress tensor $\sigma_{ij}$ is defined as:

(2) $$\sigma_{ij}=p_{h}\delta_{ij}-\eta \left(\partial_{i}u_{j}+\partial_{j}u_{i}\right)+\left(\frac{2}{3}\eta -\xi \right)\partial_{k}u_{k}\delta_{ij},$$

where η is the fluid dynamic viscosity tied to kinematic viscosity ν as η=ρν, ξ the bulk viscosity and $p_{h}$ the hydrodynamic pressure. The chemical potential $\mu_{\phi }$ is defined as

(3) $$\mu_{\phi }=2\beta \phi \left(\phi -1\right)\left(2\phi -1\right)\kappa \Delta \phi ,$$

where $\Delta =\nabla^{2}$ is the Laplacian operator, and β and κ are parameters specific to the AC formulation. The second term on the right hand side (RHS) of Equation (1) accounts for surface-tension effects. For the sake of clarity, free parameters are detailed in the next paragraph.

The interface was tracked using the conservative AC equation, where order parameter ϕ evolved as [24,25]:

(4) $$\partial_{t}\phi +\partial_{i}u_{i}\phi -\partial_{i}M\left[\partial_{i}\phi -n_{i}\frac{4\phi (1-\phi )}{W}\right]=0,$$

where the order parameter ϕ takes on values between 0 and 1, *M* is mobility, *W* is interface thickness, and $n_{i}$ is the unit normal to the interface, obtained as

(5) $$n_{i}=\frac{\partial_{i}\phi }{\left||\nabla \phi |\right|}.$$

Interfaces can be found through isosurfaces of the order parameter, i.e., ϕ=1/2. To recover the correct surface tension, free parameters appearing in the chemical potential, i.e., κ and β, are tied to surface tension σ and interface thickness *W* in the AC equation via β=12σ/W and κ=3σW/2.

### 2.2. LB Formulation for Conservative Phase-Field Equation

The conservative AC equation can be readily recovered by appropriately defining the discrete equilibrium state and relaxation coefficient in the advection–diffusion LB model:

(6) $$\partial_{t}g_{\alpha }+c_{\alpha ,i}\partial_{i}g_{\alpha }+S_{\alpha }=\Omega_{\alpha }^{\phi },$$

where $g_{\alpha }$ and $c_{\alpha }$ are populations and velocities in the discrete velocity kinetic model, and the collision operator is defined as

(7) $$\Omega_{\alpha }^{\phi }=\frac{1}{\tau_{\phi }}\left(g_{\alpha }^{\left(eq\right)}-g_{\alpha }\right).$$

The EDF is defined as

(8) $$g_{\alpha }^{\left(eq\right)}=w_{\alpha }\phi \sum_{n=0}^{2}\frac{1}{n!c_{s}^{2n}}H_{n}:a_{n}^{\left(eq\right)},$$

where $H_{n}$ and $a_{n}^{\left(eq\right)}$ are the Hermite polynomial and coefficient of order *n*, $c_{s}$ is lattice sound speed, and $w_{\alpha }$ are weights tied to each discrete velocity (resulting from the Gauss–Hermite quadrature). The expressions for these polynomials and corresponding coefficients are listed in Appendix A. The source term in Equation (6) is defined as [26]

(9) $$S_{\alpha }=w_{\alpha }H_{i}n_{i}\frac{4\phi (1-\phi )}{W}.$$

Given that the source term affects the first-order moment, a nonconserved moment of the distribution function, the distribution function is tied to the phase parameter as

(10) $$\phi =\sum_{\alpha }g_{\alpha }.$$

The relaxation coefficient is fixed as

(11) $$\tau_{\phi }=\frac{M}{c_{s}^{2}}.$$

After integration in space/time, the now-famous collision-streaming form can be recovered:

(12) $${\bar{g}}_{\alpha }\left(x+c_{\alpha }\delta_{t},t+\delta_{t}\right)=\left(1-\frac{\delta_{t}}{{\bar{\tau }}_{\phi }}\right){\bar{g}}_{\alpha }\left(x,t\right)+\frac{\delta_{t}}{{\bar{\tau }}_{\phi }}g_{\alpha }^{\left(eq\right)}\left(x,t\right)+\delta_{t}{\bar{S}}_{\alpha }\left(x,t\right),$$

where the source term takes on a new form, i.e.,

(13) $${\bar{S}}_{\alpha }=\left(1-\frac{1}{2\tau_{\phi }}\right)w_{\alpha }H_{i}n_{i}\frac{4\phi (1-\phi )}{W},$$

and:

(14) $${\bar{\tau }}_{\phi }=\tau_{\phi }+\frac{\delta_{t}}{2}.$$

The derivatives of the order parameter appearing in the various discrete time-evolution equations are computed using isotropic finite differences, i.e.,

(15) $$\partial_{i}\phi =\frac{1}{c_{s}^{2}}\sum_{\alpha }w_{\alpha }c_{\alpha ,i}\phi \left(x+c_{\alpha }\right),$$

and

(16) $$\partial_{i}^{2}\phi =\frac{2}{c_{s}^{2}}\sum_{\alpha }w_{\alpha }\left[\phi \left(x+c_{\alpha }\right)-\phi \left(x\right)\right].$$

While the present work makes use of a second-order EDF, the same macroscopic PDE, i.e., Equation (4), can also be recovered by using a first-order EDF and an additional correction term of the following form [27]:

(17) $$C_{\alpha }=\frac{w_{\alpha }}{c_{s}^{2}}H_{i}\partial_{t}\phi u_{i},$$

which as for Equation (13), postdiscretization changes into

(18) $${\bar{C}}_{\alpha }=\left(1-\frac{1}{2\tau_{\phi }}\right)\frac{w_{\alpha }}{c_{s}^{2}}H_{i}\partial_{t}\phi u_{i}.$$

Such correction terms were first introduced in the context of advection–diffusion LB solvers [28], and further extended to nonlinear equations in the same context [29]. Detailed derivation and multiscale analyses are readily available in the literature, e.g., [30].

### 2.3. LB Model for Flow Field

The flow solver kinetic model follows the low-Mach formulation used, among other sources, in [31,32,33], and is based on the original model introduced in [19]

(19) $$\partial_{t}f_{\alpha }^{{}^{\prime }}+c_{\alpha ,i}\partial_{i}f_{\alpha }^{{}^{\prime }}=\Omega_{\alpha }+\Xi_{\alpha },$$

where the collision operator is

(20) $$\Omega_{\alpha }=\frac{1}{\tau }\left({f_{\alpha }^{\left(eq\right)}}^{{}^{\prime }}-f_{\alpha }^{{}^{\prime }}\right),$$

$\Xi_{\alpha }$ is defined as

(21) $$\Xi_{\alpha }=c_{s}^{2}\left(\frac{f_{\alpha }^{\left(eq\right)}}{\rho }-w_{\alpha }\right)\left(c_{\alpha ,i}-u_{i}\right)\partial_{i}\rho +w_{\alpha }c_{s}^{2}\rho \partial_{i}u_{i}+\left(F_{b,i}+F_{s,i}\right)\left(c_{\alpha ,i}-u_{i}\right)\frac{f_{\alpha }^{\left(eq\right)}}{\rho },$$

and the relaxation coefficient τ is tied to fluid kinematic viscosity ν as

(22) $$\tau =\frac{\nu }{c_{s}^{2}}.$$

Forces $F_{b,i}$ and $F_{s,i}$ represent external body forces and surface tension, respectively, i.e.,

(23) $$F_{s,i}=\mu_{\phi }\partial_{i}\phi .$$

The modified distribution function $f_{\alpha }^{{}^{\prime }}$ is defined as

(24) $$f_{\alpha }^{{}^{\prime }}=w_{\alpha }p_{h}+c_{s}^{2}\left(f_{\alpha }-w_{\alpha }\rho \right),$$

where $f_{\alpha }$ is the classical isothermal distribution function. The modified equilibrium follows the same logic and is defined as

(25) $${f_{\alpha }^{\left(eq\right)}}^{{}^{\prime }}=w_{\alpha }p_{h}+w_{\alpha }\rho c_{s}^{2}\sum_{n=1}^{2}\frac{1}{n!c_{s}^{2n}}H_{n}:a_{n}^{\left(eq\right)}.$$

Density is tied to the order parameter as

(26) $$\rho =\rho_{l}+\left(\rho_{h}-\rho_{l}\right)\phi ,$$

where $\rho_{h}$ and $\rho_{l}$ are the densities of the heavy and light fluid, respectively. For detailed analysis of the macroscopic equations recovered by this model and the derivation of the discrete equations, interested readers are referred to [23,32]. In the context of the present study, the low-Mach model was wrapped in a moment-based formulation where postcollision populations $f_{\alpha }^{{}^{\prime }*}$ to be streamed as

(27) $${f^{{}^{\prime }}}_{\alpha }\left(x+c_{\alpha }\delta_{t},t+\delta_{t}\right)={f^{{}^{\prime }*}}_{\alpha }\left(x,t\right),$$

are computed as

(28) $${f^{{}^{\prime }*}}_{\alpha }=\rho c_{s}^{2}f_{\alpha }^{p*}+\frac{\delta_{t}}{2}\Xi_{\alpha }.$$

The postcollision preconditioned population $f_{\alpha }^{p*}$ is

(29) $$f_{\alpha }^{p*}=C^{-1}\left(I-W\right)K^{p}+C^{-1}WK^{p},$$

where C is the moment transform matrix from preconditioned populations to the target momentum space, I is the identity matrix, and W is the diagonal relaxation frequency matrix. Following [34], prior to transformation to momentum space, populations are preconditioned as

(30) $$f_{\alpha }^{p}=\frac{1}{\rho c_{s}^{2}}f_{\alpha }^{{}^{\prime }}+\frac{\delta_{t}}{2\rho c_{s}^{2}}\Xi_{\alpha }.$$

This preconditioning accomplishes two tasks, namely, normalizing the populations with density and thus eliminating the density dependence of the moments, and introducing the first half of the source term. As such, moments $K^{p}$ are computed as

(31) $$K_{\beta }^{p}=C_{\alpha \beta }f_{\alpha }^{p}.$$

The transformation from distribution function (DF)s to cumulants is carried out using the steps suggested in [35], which allows for a more efficient algorithm. The DFs are first transformed into central moments:

(32) $${\tilde{\Pi }}_{\beta }^{p}=\sum_{\alpha }{\left(c_{\alpha ,x}-u_{x}\right)}^{n_{x}}{\left(c_{\alpha ,y}-u_{y}\right)}^{n_{y}}{\left(c_{\alpha ,z}-u_{z}\right)}^{n_{z}}f_{\alpha }^{p}.$$

Here, $\beta =x^{n_{x}}y^{n_{y}}z^{n_{z}}$. The central moments are then transformed into the corresponding cumulants using the following relations:

(33a) $$\begin{array}{cc} K_{x}^{p}= & {\tilde{\Pi }}_{x}^{p}, \end{array}$$

(33b) $$\begin{array}{cc} K_{xy}^{p}= & {\tilde{\Pi }}_{xy}^{p}, \end{array}$$

(33c) $$\begin{array}{cc} K_{x^{2}}^{p}= & {\tilde{\Pi }}_{x^{2}}^{p}, \end{array}$$

(33d) $$\begin{array}{cc} K_{xy^{2}}^{p}= & {\tilde{\Pi }}_{xy^{2}}^{p}, \end{array}$$

(33e) $$\begin{array}{cc} K_{xyz}^{p}= & {\tilde{\Pi }}_{xyz}^{p}, \end{array}$$

(33f) $$\begin{array}{cc} K_{x^{2}yz}^{p}= & {\tilde{\Pi }}_{x^{2}yz}^{p}-\left[{\tilde{\Pi }}_{x^{2}}^{p}{\tilde{\Pi }}_{yz}^{p}+2{\tilde{\Pi }}_{xy}^{p}{\tilde{\Pi }}_{xz}^{p}\right], \end{array}$$

(33g) $$\begin{array}{cc} K_{x^{2}y^{2}}^{p}= & {\tilde{\Pi }}_{x^{2}y^{2}}^{p}-\left[{\tilde{\Pi }}_{x^{2}}^{p}{\tilde{\Pi }}_{y^{2}}^{p}+2{\left({\tilde{\Pi }}_{xy}^{p}\right)}^{2}\right], \end{array}$$

(33h) $$\begin{array}{cc} K_{xy^{2}z^{2}}^{p}= & {\tilde{\Pi }}_{xy^{2}z^{2}}^{p}-\left[{\tilde{\Pi }}_{z^{2}}^{p}{\tilde{\Pi }}_{xy^{2}}^{p}+{\tilde{\Pi }}_{y^{2}}^{p}{\tilde{\Pi }}_{xz^{2}}^{p}+4{\tilde{\Pi }}_{yz}^{p}{\tilde{\Pi }}_{xyz}^{p}+2\left({\tilde{\Pi }}_{xz}^{p}{\tilde{\Pi }}_{y^{2}z}^{p}+{\tilde{\Pi }}_{xy}^{p}{\tilde{\Pi }}_{yz^{2}}^{p}\right)\right],\\ K_{x^{2}y^{2}z^{2}}^{p}= & {\tilde{\Pi }}_{x^{2}y^{2}z^{2}}^{p}-\left[4{\left({\tilde{\Pi }}_{xyz}^{p}\right)}^{2}+{\tilde{\Pi }}_{x^{2}}^{p}{\tilde{\Pi }}_{y^{2}z^{2}}^{p}+{\tilde{\Pi }}_{y^{2}}^{p}{\tilde{\Pi }}_{x^{2}z^{2}}^{p}+{\tilde{\Pi }}_{z^{2}}^{p}{\tilde{\Pi }}_{x^{2}y^{2}}^{p}+4({\tilde{\Pi }}_{xy}^{p}{\tilde{\Pi }}_{x^{2}yz}^{p}+\right.\\ \; & \left.{\tilde{\Pi }}_{xz}^{p}{\tilde{\Pi }}_{xy^{2}z}^{p}+{\tilde{\Pi }}_{xy}^{p}{\tilde{\Pi }}_{xyz^{2}}^{p}+2\left({\tilde{\Pi }}_{xy^{2}}^{p}{\tilde{\Pi }}_{xz^{2}}^{p}+{\tilde{\Pi }}_{x^{2}y}^{p}{\tilde{\Pi }}_{yz^{2}}^{p}+{\tilde{\Pi }}_{x^{2}z}^{p}{\tilde{\Pi }}_{y^{2}z}^{p}\right))+\right. \end{array}$$

(33i) $$\begin{array}{cc} \; & \left.(16{\tilde{\Pi }}_{xy}^{p}{\tilde{\Pi }}_{xz}^{p}{\tilde{\Pi }}_{yz}^{p}+4\left({\left({\tilde{\Pi }}_{xz}^{p}\right)}^{2}{\tilde{\Pi }}_{y^{2}}^{p}+{\left({\tilde{\Pi }}_{yz}^{p}\right)}^{2}{\tilde{\Pi }}_{x^{2}}^{p}+{\left({\tilde{\Pi }}_{xy}^{p}\right)}^{2}{\tilde{\Pi }}_{z^{2}}^{p}\right)+2{\tilde{\Pi }}_{x^{2}}^{p}{\tilde{\Pi }}_{y^{2}}^{p}{\tilde{\Pi }}_{z^{2}}^{p})\right]. \end{array}$$

The remainder of the moments can be easily obtained via permutation of the indices. The collision process was performed in cumulant space according to [35]. The fluid viscosity is controlled via the collision factor related to second-order cumulants (e.g., $K_{xy}^{p}$, $K_{x^{2}}^{p}-K_{y^{2}}^{p}$, $K_{x^{2}}^{p}-K_{z^{2}}^{p}$ etc.). The rest of the collision factors were set to unity for simplicity. Once the collision step had been applied, cumulants were transformed back into central moments as

(34a) $$\begin{array}{cc} {\tilde{\Pi }}_{x}^{p*}= & K_{x}^{p*}, \end{array}$$

(34b) $$\begin{array}{cc} {\tilde{\Pi }}_{xy}^{p*}= & K_{xy}^{p*}, \end{array}$$

(34c) $$\begin{array}{cc} {\tilde{\Pi }}_{x^{2}}^{p*}= & K_{x^{2}}^{p*}, \end{array}$$

(34d) $$\begin{array}{cc} {\tilde{\Pi }}_{xy^{2}}^{p*}= & K_{xy^{2}}^{p*}, \end{array}$$

(34e) $$\begin{array}{cc} {\tilde{\Pi }}_{xyz}^{p*}= & K_{xyz}^{p*}, \end{array}$$

(34f) $$\begin{array}{cc} {\tilde{\Pi }}_{x^{2}yz}^{p*}= & K_{x^{2}yz}^{p*}+\left[{\tilde{\Pi }}_{x^{2}}^{p*}{\tilde{\Pi }}_{yz}^{p*}+2{\tilde{\Pi }}_{xy}^{p*}{\tilde{\Pi }}_{xz}^{p*}\right], \end{array}$$

(34g) $$\begin{array}{cc} {\tilde{\Pi }}_{x^{2}y^{2}}^{p*}= & K_{x^{2}y^{2}}^{p*}+\left[{\tilde{\Pi }}_{x^{2}}^{p*}{\tilde{\Pi }}_{y^{2}}^{p*}+2{\left({\tilde{\Pi }}_{xy}^{p*}\right)}^{2}\right], \end{array}$$

(34h) $$\begin{array}{cc} {\tilde{\Pi }}_{xy^{2}z^{2}}^{p*}= & K_{xy^{2}z^{2}}^{p*}+\left[{\tilde{\Pi }}_{z^{2}}^{p*}{\tilde{\Pi }}_{xy^{2}}^{p*}+{\tilde{\Pi }}_{y^{2}}^{p*}{\tilde{\Pi }}_{xz^{2}}^{p*}+4{\tilde{\Pi }}_{yz}^{p*}{\tilde{\Pi }}_{xyz}^{p*}+2\left({\tilde{\Pi }}_{xz}^{p*}{\tilde{\Pi }}_{y^{2}z}^{p*}+{\tilde{\Pi }}_{xy}^{p*}{\tilde{\Pi }}_{yz^{2}}^{p*}\right)\right],\\ {\tilde{\Pi }}_{x^{2}y^{2}z^{2}}^{p*}= & K_{x^{2}y^{2}z^{2}}^{p*}+\left[4{\left({\tilde{\Pi }}_{xyz}^{p*}\right)}^{2}+{\tilde{\Pi }}_{x^{2}}^{p*}{\tilde{\Pi }}_{y^{2}z^{2}}^{p*}+{\tilde{\Pi }}_{y^{2}}^{p*}{\tilde{\Pi }}_{x^{2}z^{2}}^{p*}+{\tilde{\Pi }}_{z^{2}}^{p*}{\tilde{\Pi }}_{x^{2}y^{2}}^{p*}+4({\tilde{\Pi }}_{xy}^{p*}{\tilde{\Pi }}_{x^{2}yz}^{p*}+\right.\\ \; & \left.{\tilde{\Pi }}_{xz}^{p*}{\tilde{\Pi }}_{xy^{2}z}^{p*}+{\tilde{\Pi }}_{xy}^{p*}{\tilde{\Pi }}_{xyz^{2}}^{p*}+2\left({\tilde{\Pi }}_{xy^{2}}^{p*}{\tilde{\Pi }}_{xz^{2}}^{p*}+{\tilde{\Pi }}_{x^{2}y}^{p*}{\tilde{\Pi }}_{yz^{2}}^{p*}+{\tilde{\Pi }}_{x^{2}z}^{p*}{\tilde{\Pi }}_{y^{2}z}^{p*}\right))-\right. \end{array}$$

(34i) $$\begin{array}{cc} \; & \left.(16{\tilde{\Pi }}_{xy}^{p*}{\tilde{\Pi }}_{xz}^{p*}{\tilde{\Pi }}_{yz}^{p*}+4\left({\left({\tilde{\Pi }}_{xz}^{p*}\right)}^{2}{\tilde{\Pi }}_{y^{2}}^{p*}+{\left({\tilde{\Pi }}_{yz}^{p*}\right)}^{2}{\tilde{\Pi }}_{x^{2}}^{p*}+{\left({\tilde{\Pi }}_{xy}^{p*}\right)}^{2}{\tilde{\Pi }}_{z^{2}}^{p*}\right)+2{\tilde{\Pi }}_{x^{2}}^{p*}{\tilde{\Pi }}_{y^{2}}^{p*}{\tilde{\Pi }}_{z^{2}}^{p*})\right]. \end{array}$$

After this step, postcollision central moments could be readily transformed back into populations. All transforms presented here and upcoming simulations are based on the D3Q27 stencil. The following set of 27 moments were used as the basis for the moments:

(35) $$\begin{array}{c} \beta \in \{0,x,y,z,xy,xz,yz,x^{2}-y^{2},x^{2}-z^{2},x^{2}+y^{2}+z^{2},\\ xy^{2}+xz^{2},xyz,xy^{2}-xz^{2},x^{2}+yz^{2},x^{2}z+y^{2}z,x^{2}y-yz^{2},x^{2}z-y^{2}z,x^{2}y^{2}-2x^{2}z^{2}+y^{2}z^{2},\\ x^{2}y^{2}+x^{2}z^{2}-2y^{2}z^{2},x^{2}y^{2}+x^{2}z^{2}+y^{2}z^{2},x^{2}yz,xy^{2}z,xyz^{2},x^{2}y^{2}z,x^{2}yz^{2},xy^{2}z^{2},x^{2}y^{2}z^{2}\}, \end{array}$$

where $\beta =x^{2}-y^{2}$ stands for a central moment of form ${\tilde{\Pi }}_{x^{2}}^{p}-{\tilde{\Pi }}_{y^{2}}^{p}$. Previous systematic studies of the flow solver showed second-order convergence under diffusive scaling [32].

## 3. Numerical Applications

In this section, the proposed numerical method is validated through different test cases. All results and simulation parameters are reported in LB units, i.e., nondimensionalized with time step, grid size, and heavy fluid density.

### 3.1. Static Droplet: Surface-Tension Measurement

**Figure 1:**
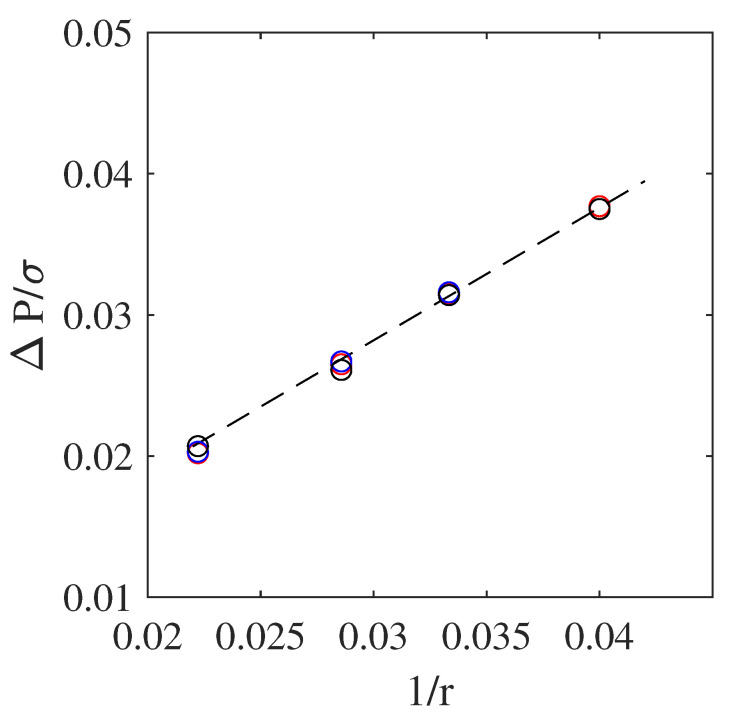
Changes in pressure difference around droplet for different surface tensions and droplet radii. Red, blue, and black symbols illustrate results from present study with $\sigma =10^{-1},10^{-3}$, and $10^{-6}$, respectively.

As a first test, to validate the hydrodynamics of the model, we considered the case of a static droplet in a rectangular domain with periodic boundaries all around. All cases consisted of a domain of 256×256 size filled with a light fluid. A droplet of the heavier fluid was placed at the center of the domain. Simulations were pursued till the system had converged. The pressure difference between the droplet and surrounding lighter fluid was then extracted. Using Laplace’s law, i.e.,

(36) $$\Delta P=\frac{\sigma }{r},$$

where ΔP is the pressure difference, and *r* the droplet radius, one can readily obtain the effective surface tension. Three different surface tensions, i.e., $\sigma =1\times 10^{-1}$, $1\times 10^{-3}$, and $1\times 10^{-6}$, along with four different droplet radii, i.e., r=25, 30, 35, and 45, were considered here. Obtained results are shown in Figure 1. Results presented here consider a density ratio of 20 and nondimensional viscosity of 0.1.

The model satisfied Laplace’s law and recovered the correct surface tensions. Furthermore, it could span a wide range of surface tensions, as opposed to other classes of multiphase solvers, such as free energy or pseudopotential formulations [36,37], and maintain relatively low spurious currents. For example, at a density ratio of 1000 and $\sigma =10^{-3}$, spurious currents were found to be only of the order of $10^{-6}$, in strong contrast with previously cited approaches.

### 3.2. Rayleigh–Taylor Instability

The Rayleigh–Taylor instability is a well-known and widely studied gravity-driven effect occurring when a layer of a heavier fluid lies on top of another layer of a lighter fluid [38,39,40]. Perturbation at the interface between the two fluids causes the heavier one to penetrate the lighter fluid. In general, the dynamics of this system are governed by two nondimensional parameters, namely, the Atwood and Reynolds numbers. The former is defined as

(37) $$\mathrm{At}=\frac{\rho_{h}-\rho_{l}}{\rho_{h}+\rho_{l}},$$

while the latter is:

(38) Re=ρhU*Lμh,

where $\rho_{l}$ and $\rho_{h}$ are densities of the heavy and light fluids, respectively, $\mu_{h}$ is the dynamic viscosity of the heavy fluid, $L_{x}$ the size of the domain in the horizontal direction and $U^{*}$ the characteristic velocity, defined as

(39) $$U^{*}=\sqrt{gL_{x}},$$

where *g* is gravity-driven acceleration. The characteristic time for this case is defined as

(40) $$T=\frac{L_{x}}{U^{*}}.$$

Following the setup studied in [19], we considered a domain sized $L_{x}\times 4L_{x}$ with $L_{x}=600$. Initially, the top half of the domain was filled with the heavy liquid, and the bottom half with the lighter one. The interface was perturbed via the following profile:

(41) $$h_{i}\left(x\right)=\frac{L}{10}\cos\left(\frac{2\pi x}{L_{x}}\right)+2L_{x}.$$

**Figure 2:**
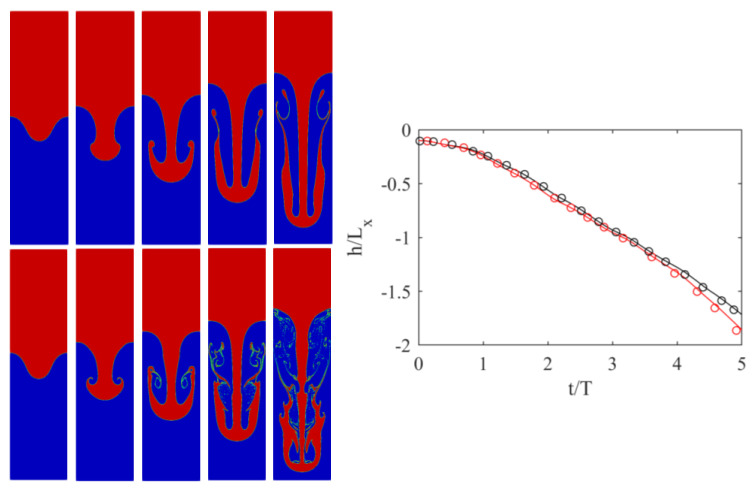
(**Left**) Evolution of interface for Rayleigh–Taylor instability for (**top row**) Re = 256 and (**bottom row**) Re = 2048 at different times: (from **left** to **right**) t/T=1, 2, 3, 4, and 5. (**Right**) Position of penetrating spike over time: (black) Re = 256 and (red) Re = 2048. (plain lines) Results and (symbols) data from [19].

While periodic boundaries were applied in the horizontal direction, at the top and bottom boundaries, no-slip boundary conditions were applied using the half-way bounce-back scheme [1]. The At number was set to 0.5, while two different Re numbers were considered, i.e., Re = 256 and 2048. In both cases, $g=6\times 10^{-6}$, while the nondimensional viscosities were 0.1406 and 0.0176, respectively. To validate the simulations, the position of the downward-plunging heavy liquid spike was measured over time and compared to the reference data from [19]. Results are illustrated in Figure 2.

**Figure 3:**
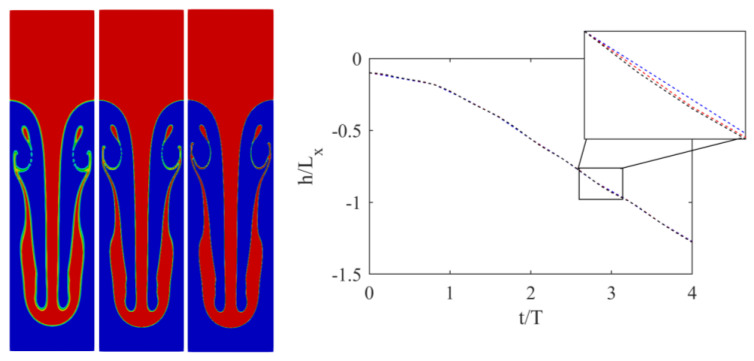
(**Left**) Interface for Rayleigh–Taylor instability at t/T= 5 and Re = 256 for three different resolutions (**left** to **right**) $L_{x}$ = 150, 300, and 600. (**Right**) Position of penetrating spike over time: (black) $L_{x}$ = 600, (red) $L_{x}$ = 300, and (blue) $L_{x}$ = 150.

**Figure 4:**
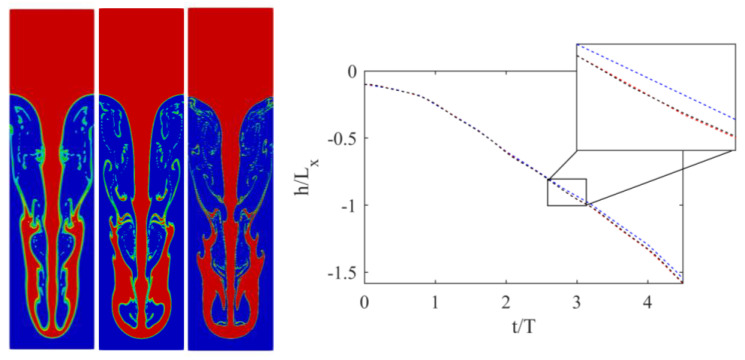
(**Left**) Interface for Rayleigh–Taylor instability at t/T = 5 and Re = 2048 for three different resolutions (**left** to **right**) $L_{x}$ = 150, 300, and 600. (**Right**) Position of penetrating spike over time: (black) $L_{x}$ = 600, (red) $L_{x}$ = 300, and (blue) $L_{x}$ = 150.

Both simulations agreed very well with the reference solution of [19]. To showcase the ability of the solver to handle under-resolved simulations, and illustrate the convergence of the obtained solutions, simulations were repeated at two additional lower resolutions with $L_{x}$ = 300 and 150, with an acoustic scaling of the time-step size. Results obtained with those lower resolutions are shown in Figure 3 and Figure 4.

The position of the plunging spike clearly shows that, while minor differences exist, even the lowest resolution captures the correct position. Smaller features, however, especially at Re = 2048, need higher resolutions to be correctly captured. At Re = 256 for instance, even the secondary instability was converged as, at $L_{x}$ = 300, no segmentation was observed. For Re = 2056, on the other hand, while a larger structure started to converge, thinner features clearly needed more resolutions.

### 3.3. Turbulent 3D Rayleigh–Taylor Instability

To further showcase the ability of the solver to deal with complex flows, we also considered the Rayleigh–Taylor instability in 3D. The studied configuration followed those studied in [41]. The definitions of nondimensional parameters were similar to those used in the previous section. The domain was discretized using 100×100×1200 grid points, with L=100. The interface was placed at the center of the domain along the *z* axis and perturbed using

(42) $$h_{i}\left(x,y\right)=\frac{L}{10}\left[\cos\left(\frac{2\pi x}{L}\right)+\cos\left(\frac{2\pi y}{L}\right)\right]+6L,$$

and Reynolds and Atwood numbers were set to 1000 and 0.15, respectively. As for previous configurations, periodic boundaries were applied in the horizontal direction and no-slip boundaries at the top and bottom. The body force was set to $g=3.6\times 10^{-5}$, and viscosity to 0.006. The position of the downward-plunging spike was measured over time and compared to reference data from [41]. After the penetration of the two liquids into each other, the Kelvin–Helmholtz instability caused the plunging spike to roll up and take a mushroomlike shape. As the mushroom-shaped spike further progressed into the lighter fluid, the cap disintegrated into four fingerlike structures. As is shown later, these fingers were reminiscent of instabilities leading to splashing in the impact of a droplet on liquid surfaces.

**Figure 5:**
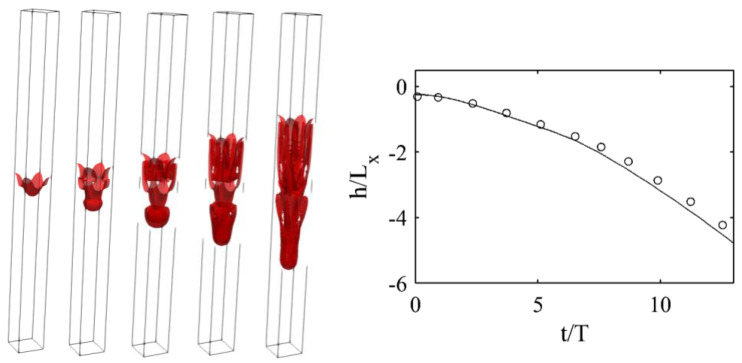
(**Left**) Evolution of interface for 3D Rayleigh–Taylor instability for Re = 1000 at different times: (from **left** to **right**) t/T = 1.9, 3.9, 5.8, 7.8, and 9.7. (**Right**) Position of penetrating spike over time: (plain lines) Results and (symbols) data from [41].

Overall, as shown in Figure 5, obtained results from the present simulation were in good agreement with the reference data.

### 3.4. Droplet Splashing on Thin Liquid Film

**Figure 6:**
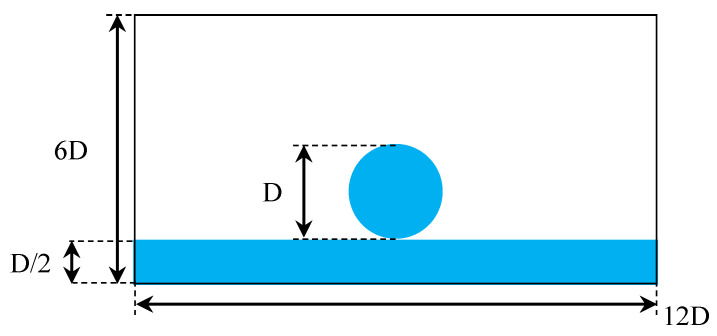
Geometrical configuration of droplet impact on liquid sheet case in 2D.

As the final case, we considered the impact of a droplet on a thin liquid layer. This configuration is interesting, as it involves complex dynamics such as splashing, and it is of interest in many areas of science and engineering [42,43]. Immediately after impact, the liquid surface is perturbed. In many instances, at the contact point (line), a thin liquid jet forms, and it continues to grow and propagate as a corolla. As the crownlike structure radially propagates, a rim starts to form. At high-enough Weber numbers, the structure breaks into small droplets via the Rayleigh–Plateau instability [44]. A detailed study of the initial stages of the spreading process showed that the spreading radius scales with time regardless of Weber and Reynolds numbers [44]. While widely studied in the literature using different numerical formulations [26,45,46,47], simulations are usually limited to lower density and viscosity ratios, and/or Weber and Reynolds numbers [26,36,45,46]. As such, we first focused on a 2D configuration considering three sets of We and Re numbers, namely: Re = 200 and We = 220, Re = 1000 and We = 220 and Re = 1000 and We = 2200. In all simulations, density and viscosity ratios were set to $\rho_{h}/\rho_{l}=1000$ and $\nu_{l}/\nu_{h}$ = 15, emulating a water/air system. The geometrical configuration is illustrated in Figure 6.

The top- and bottom-boundary conditions were set to walls modelled with the half-way bounce-back formulation, while symmetrical boundaries were applied to the left and right. The droplet diameter was resolved with 100 grid points. Initial velocity in the droplet was set to $U_{0}=$0.05, and $\nu_{L}$ was determined via the Reynolds number:

(43) Re=ρhU0Dμh.

Furthermore, the We number is defined as

(44) $$\mathrm{We}=\frac{\rho_{l}D{U_{0}}^{2}}{\sigma }.$$

**Figure 7:**
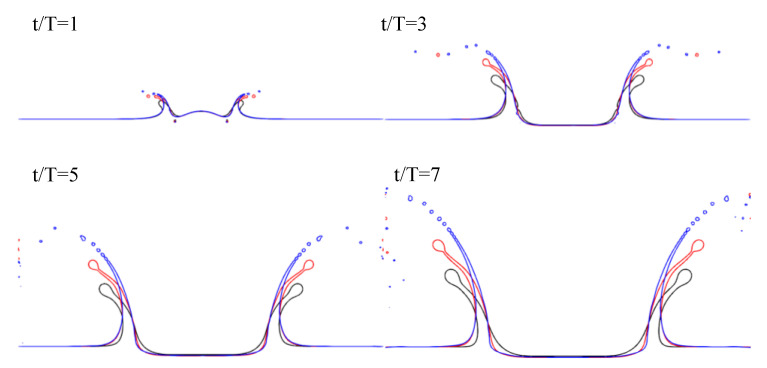
Impact of circular droplet on liquid sheet at different We and Re numbers with $\rho_{h}/\rho_{l}=1000$ and $\nu_{l}/\nu_{h}=15$. (black) Re = 200 and We = 220, (red) Re = 1000 and We = 220, and (blue) Re = 1000 and We = 2200.

The evolution of the liquid surface, as obtained from the simulations, is shown in Figure 7. Following [44], rim breakup and splashing occurred for larger impact parameters, defined as

(45) K=We1/2Re1/4.

Accordingly, impact parameters for the studied 2D cases were K = 55.7, 83.4, and 263.8. The evolution of the systems in Figure 7 clearly shows that, in agreement with observations in [44], larger values of the impact parameter led to droplet detachment from the rim and splashing.

**Figure 8:**
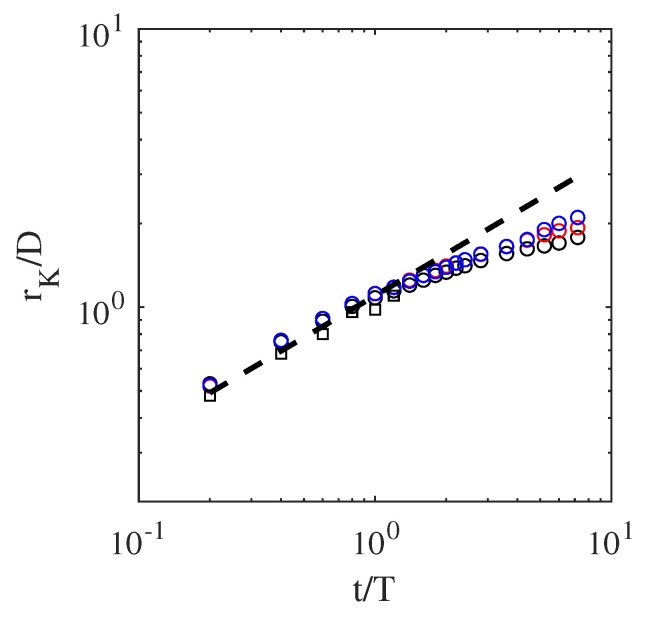
Evolution of spreading radius $r_{K}$ as function of time for droplet impact on liquid film case. Circular symbols designate 2D simulations: (black) Re = 200 and We = 220, (red) Re = 1000 and We = 220, and (blue) Re = 1000 and We = 2200. Rectangular symbols belong to 3D simulation with Re = 1000 and We = 8000. Dashed line is $\frac{r_{K}}{D}=1.1\sqrt{t/T}$.

Furthermore, the evolution of spreading radii $r_{K}$ over time for different cases is shown in Figure 8. The radii scaled with time at the initial stages of the impact, in agreement with results reported in [44].

**Figure 9:**
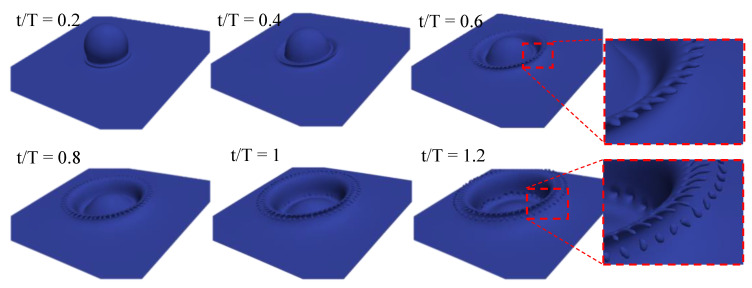
Impact of spherical droplet on thin liquid sheet at We = 8000 and Re = 1000 at different times with $\rho_{h}/\rho_{l}=1000$ and $\nu_{l}/\nu_{h}=15$.

As a final test case, to showcase the robustness of the proposed algorithm, a 3D configuration with Re = 1000 and We = 8000 was also ran. The evolution of the liquid surface over time is shown in Figure 9.

After the initial impact, a thin liquid jet was formed at the contact line between the droplet and sheet. Then, the crown evolved and spread. At later stages, the fingerlike structures started to form at the tip of the crown. These liquid fingers then became detached from the crown, and liquid splashing was observed. This sequence of events was in excellent agreement with those presented in [44]. Furthermore, the spreading radius, as plotted in Figure 8, agreed with the theoretical predictions.

## 4. Conclusions

An LB-based solver relying on the conservative AC equation, and a modified hydrodynamic pressure/velocity-based distribution and MRT collision operator in cumulant space was presented in this study with the aim to model multiphase flows in larger Weber/Reynolds regimes. While stability at high Weber numbers, i.e., low surface tensions, is achieved through the decoupled nature of conservative AC formulation, the added stability in terms of kinematic viscosity, i.e., larger Reynolds numbers, is brought about by the collision operator and modified pressure-based LB formulation for the flow. Compared to other models available in the literature based on AC formulation, the use of cumulants allows for stability at considerably higher Reynolds numbers, i.e., lower values of the relaxation factor. For instance, configurations such as 3D droplet splashing were not stable with single relaxation time (SRT) formulation for the same choice of nondimensional parameters, i.e., resolution and relaxation factor. The algorithm was shown to capture flow dynamics and be stable in the targeted regimes. The application of the proposed algorithm to more complex configurations, such as liquid jets, is currently being studied and will be reported in future publications. 

## Data Availability

The data presented in this study are available on request from the corresponding author.

## Appendix A. Hermite Polynomials and Coefficients

Hermite polynomials used in EDFs of different solvers, defined as

(A1a) $$\begin{array}{cc} H_{0} & =1, \end{array}$$

(A1b) $$\begin{array}{cc} H_{i} & =c_{\alpha ,i}, \end{array}$$

(A1c) $$\begin{array}{cc} H_{ij} & =c_{\alpha ,i}c_{\alpha ,j}-c_{s}^{2}\delta_{ij}, \end{array}$$

where $\delta_{ij}$ denotes Kronecker delta function, while corresponding equilibrium coefficients are

(A2a) $$\begin{array}{cc} a_{0}^{\left(eq\right)} & =\rho , \end{array}$$

(A2b) $$\begin{array}{cc} a_{i}^{\left(eq\right)} & =\rho u_{i}, \end{array}$$

(A2c) $$\begin{array}{cc} a_{ij}^{\left(eq\right)} & =\rho u_{i}u_{j}, \end{array}$$
